# Immunological characteristics of human umbilical cord mesenchymal stem cells and the therapeutic effects of their transplantion on hyperglycemia in diabetic rats

**DOI:** 10.3892/ijmm.2013.1572

**Published:** 2013-11-28

**Authors:** HONGWU WANG, XIAOYAN QIU, PING NI, XUERONG QIU, XIAOBO LIN, WEIZHAO WU, LICHUN XIE, LIMIN LIN, JUAN MIN, XIULAN LAI, YUNBIN CHEN, GUYU HO, LIAN MA

**Affiliations:** 1 Department of Pediatrics, Second Affiliated Hospital of Shantou University Medical College, Shantou, Guangdong 515041, P.R. China; 2 Transformation Medical Center, Second Affiliated Hospital of Shantou University Medical College, Shantou, Guangdong 515041, P.R. China; 3 Department of Pediatrics, Guangdong Women’s And Children’s Hospital, Guangzhou, Guangdong 510010, P.R. China; 4 Cancer Hospital of Shantou University Medical College, Shantou, Guangdong 515041, P.R. China; 5 Department of Obstetrics and Gynecology, Shenzhen Pingshan Women’s And Children’s Hospital, Shenzhen, Guangdong 518118, P.R. China

**Keywords:** umbilical cord, mesenchymal stem cells, hyperglycemic, transplantation, immunological characteristics

## Abstract

Islet transplantation involves the transplantation of pancreatic islets from the pancreas of a donor to another individual. It has proven to be an effective method for the treatment of type 1 diabetes. However, islet transplantation is hampered by immune rejection, as well as the shortage of donor islets. Human umbilical cord Wharton’s jelly-derived mesenchymal stem cells (HUMSCs) are an ideal cell source for use in transplantation due to their biological characteristics and their use does not provoke any ethical issues. In this study, we investigated the immunological characteristics of HUMSCs and their effects on lymphocyte proliferation and the secretion of interferon (IFN)-γ, and explored whether direct cell-to-cell interactions and soluble factors, such as IFN-γ were important for balancing HUMSC-mediated immune regulation. We transplanted HUMSCs into diabetic rats to investigate whether these cells can colonize *in vivo* and differentiate into pancreatic β-cells, and whether the hyperglycemia of diabetic rats can be improved by transplantation. Our results revealed that HUMSCs did not stimulate the proliferation of lymphocytes and did not induce allogeneic or xenogeneic immune cell responses. qRT-PCR demonstrated that the HUMSCs produced an immunosuppressive isoform of human leukocyte antigen (HLA-I) and did not express HLA-DR. Flow cytometry revealed that the HUMSCs did not express immune response-related surface antigens such as, CD40, CD40L, CD80 and CD86. IFN-γ secretion by human peripheral blood lymphocytes was reduced when the cells were co-cultured with HUMSCs. These results suggest that HUMSCs are tolerated by the host in an allogeneic transplant. We transplanted HUMSCs into diabetic rats, and the cells survived in the liver and pancreas. Hyperglycemia of the diabetic rats was improved and the destruction of pancreatic cells was partly repaired by HUMSC transplantation. Hyperglycemic improvement may be related to the immunomodulatory effects of HUMSCs. However, the exact mechanisms involved remain to be further clarified.

## Introduction

Type 1 diabetes is an insulin-dependent, autoimmune disorder characterized by the destruction of insulin-producing β-cells (1). Islet transplantation involves the transplantation of pancreatic islets from the pancreas of a donor to another individual. It has proven to be an effective method for the treatment of type 1 diabetes, as well as for patients with diabetic nephropathy, retinopathy and other complications (2,3). However, successful islet transplantation is hampered by immune rejection, as well as the shortage of donor islets (4). Stem cells possess the ability to differentiate into functional insulin-producing cells (5), suggesting that these cells are a promising source for obtaining a sufficient number of islet cells.

Studies have indicated that bone marrow mesenchymal stem cells (BMSCs) (6) and embryonic stem cells (ESCs) (7) can differentiate into insulin-producing cells and can used in transplantion therapy for type 1 diabetes. However, ESCs can not be widely applied in clinical practice due to the ethical issues that are provoked by their use. To date, the study of mesenchymal stem cells (MSCs) has mainly focused on BMSCs. However, the invasiveness of the bone marrow aspiration procedure and the age-dependent degradation of the quantity and quality of BMSCs limit their clinical potential (8,9).

Human umbilical cord Wharton’s jelly is a new source of MSCs that exhibit a high degree of self-renewal capacity and multi-differentiation potential. Human umbilical cord Wharton’s jelly-derived mesenchymal stem cells (HUMSCs) have a wider range of collection sources than BMSCs (10) or ESCs (11), and can be easily collected with fewer ethical constraints. As an alternative source of MSCs, HUMSCs have promising clinical application prospects. However, the immune rejection problems associated with their use need to be solved before they can be considered for successful transplantation. AS previously demonstrated, MSCs can suppress lymphocyte proliferation induced by phytohemagglutinin (PHA) and that these cells are not restricted by major histocompatibility complex (MHC) (12). An allograft has been demonstrated to minimize the risk of rejection following transplantation, even between unmatched individuals (12). These unique immunological properties of MSCs increase their potential for use in the organ transplantion and for the prevention of rejection, as well as for the treatment of autoimmune disease.

Although the immunogenic behavior of BMSCs has been characterized (13,14), the immunoregulatory properties of HUMSCs have not been fully defined. Interferon-γ (IFN-γ) activates and promotes lymphocyte function as a positive immune regulator in immune rejection. In this study, we investigated the immunological characteristics of HUMSCs and their effects on lymphocyte proliferation and the secretion of IFN-γ, and explored whether direct cell-to-cell interactions and soluble factors, such as IFN-γ are important for balancing HUMSC-mediated immune regulation. We transplanted HUMSCs into diabetic rats to determine whether these cells can colonize *in vivo* and differentiate into pancreatic β-cells, and examined whether the hyperglycemia of diabetic rats can be improved by HUMSC transplantation.

## Materials and methods

### Cell culture

Ethical approval was obtained from the Institutional Review Board of Shantou University Medical College, Shantou, China. Human umbilical cords from consenting patients (full-term caesarian sections) were collected immediately into a sterilized 50 ml tube, washed with phosphate-buffered saline (PBS) and cut into small 2–3-cm-thick sections. After dissecting the arteries and veins, the remaining tissue, the Wharton’s jelly, was diced into smaller fragments and transferred to a 75 cm^2^ flask in DMEM/F12 (Sigma-Aldrich, St. Louis, MO, USA) culture medium supplemented with 10% fetal bovine serum (FBS; Gibco, Sydney, Australia), 100 μg/ml penicillin/streptomycin (Shanghai Bioscience, Shangai, China), 1 g/ml amphotericin B (Gilead Sciences, Inc., San Dimas, CA, USA), 5 ng/ml epidermal growth factor (EGF; Invitrogen Life Technologies, Carlsbad, CA, USA) and 5 ng/ml basic fibroblast growth factor (bFGF; Sigma-Aldrich). The cultures were left undisturbed for 5–7 days at 37°C, 5% CO_2_ to allow the migration of cells from the explants, after which the medium was replaced.

### Phenotypic characterization of HUMSCs

Approximately 1×10^6^ HUMSCs at passage 3 were dispersed with trypsin and resuspended in PBS containing phycoerythrin (PE)-conjugated antibodies against CD40, CD40L, CD80 and CD86 (BD Biosciences, Franklin Lakes, NJ, USA) for 60 min at 4°C. The cells were washed 3 times with PBS and incubated with PE-conjugated rabbit anti-mouse IgG (Santa Cruz Biotechnology, Inc., Santa Cruz, USA) or FITC-conjugated goat anti-rat IgG (Santa Cruz Biotechnology) for 30 min at room temperature. After 3 washes, the cells were resuspended in 0.5 ml PBS and analyzed by flow cytometry with the use of Epics XL flow cytometer (Beckman Coulter, Brea, CA, USA).

### Lymphocyte proliferation assay

Human peripheral blood lymphocytes (PBMCs) were isolated from healthy donors by Ficoll-Paque (1.077 g/ml) density gradient centrifugation. The cell concentration was adjusted to 1×10^6^/ml with RPMI-1640 medium (Gibco, Carlsbad, CA, USA) supplemented with 10% FBS. HUMSCs at passage 3 were harvested and adjusted to 1×10^3^ cells/ml, 1×10^4^ cells/ml, or 1×10^5^ cells/ml in L-DMEM containing 10% FBS. A 100 μl suspension of HUMSCs was plated into 96-well plates. The plates were incubated for 72 h at 37°C, 5% CO_2_. After the cells reached 70–80% confluence, the medium was removed and 100 μl of fresh medium containing 2.5 μl of mitomycin C (1 μg/μl; Sigma-Aldrich) were added for 30 min at 37°C to mitotically inactivate the HUMSCs. After the medium was removed, the inactivated HUMSCs were washed twice with PBS. HUMSCs were resuspended in 100 μl of lymphocyte medium (RPMI-1640 containing 10% FBS), co-cultured with 1×10^5^ cells/l PBMC, and stimulated by PHA (10 mg/l) (Sigma-Aldrich) for 72 h at 37°C, 5% CO_2_. The cells were divided into the following groups: PBMCs + PHA (positive control); HUMSCs (1×10^5^) + PBMCs + PHA; HUMSCs (1×10^4^) + PBMCs + PHA; and HUMSCs (1×10^3^) + PBMCs + PHA. Three ratios of HUMSCs to PBMCs were used: 1:1, 1:10 and 1:100. Each trial was repeated in triplicate. The CCK-8 kit (Dojindo Molecular Technologies, Inc., Kumamoto, Japan) was used to assess the immunomodulatory impact of HUMSCs on PBMCs following stimulation with PHA. The procedure was carried out according to the manufacturer’s instructions. The inhibitory effects of HUMSCs on lymphocyte proliferation were evaluated by comparing the optical density (OD) in cells co-cultured with inactivated HUMSCs with the OD of lymphocytes cultured alone.

### ELISA

Passage 3 HUMSCs were trypsinized, the concentrations adjusted to 1×10^5^ cells/ml, and the cells were plated (1 ml) in 24-well plates. After the cells reached 70% confluence, 10 μl mitomycin-C (1 μg/μl) were added into each well. Following incubation for 1 h at 37°C, 5% CO_2_, the medium was removed and the cells were washed twice with PBS. PBMCs (1×10^5^) in 1 ml of lymphocyte medium were added and co-cultured in the presence of 10 μl of PHA for 72 h at 37°C, 5% CO_2_. The groups were as follows: PBMCs + PHA (positive control); unstimulated PBMCs (negative control); and HUMSCs (1×10^5^ cells) + PBMCs + PHA. The supernatants of each group were collected and IFN-γ expression was evaluated using the ELISA detection kit according to the manufacturer’s instructions (Invitrogen).

### Quantitative reverse transcription-polymerase chain reaction (qRT-PCR)

Total RNA was isolated from the HUMSCs. In addition, we extracted the pancreas of the diabetic rats after HUMSC transplantation, as well as the pancreas of diabetic rats without HUMSC transplantation using TRIzol reagent (Invitrogen) according to the manufacturer’s instructions. cDNA was prepared using the Prime Script RT Reagent kit (Takara Bio, Inc., Shiga, Japan). cDNA samples were analyzed by quantitative PCR using SYBR premix (Takara) in an ABI 7300 system. The primers used for qRT-PCR analyses were as follows: human pancreatic and duodenal homeobox 1 (PDX1; 148 bp) forward, 5′-ttcacgagccagtatgaccttcac-3′ and reverse, 5′-gaagacagacctgggatgcaca-3′; human insulin (221 bp) forward, 5′-acccagccgcagcctttgtg-3′ and reverse, 5′-ttccacaatgcc acgcttctgc-3′; human glucagon (161 bp) forward, 5′-cagagctta ggacacagagcacatc-3′ and reverse, 5′-acgttgccagctgccttgta-3′; HLA-I (293 bp) forward, 5′-gcagacacggaatgtgaagg-3′ and reverse, 5′-gtaggctctcaactgctccg-3′; HLA-DR (350 bp) forward, 5′-tcttgtctgttctgcctcactc-3′ and reverse, 5′-ttccaggttggctttgtcc-3′; and β-actin (396 bp) forward, 5′-tggcaccacaccttctacaatgagc-3′ and reverse, 5′-gcacagcttctccttaatgtcacgc-3′.

### Adenoviral expansion and infection

The E1-deleted adenovirus (serotype 5) carrying the CMV promoter/EGFP hybrid gene was purchased from Vector Gene Technology Company (Beijing, China). For amplification of the adenoviruses, 1×10^8^ infection units/ml (IU/ml) of viruses was added into a 10-cm dish pre-seeded with 1×10^6^ Ad293 cells (Stratagene, La Jolla, CA, USA) overnight. Following incubation for 30–48 h, the cells were harvested by scraping and centrifugation at 3,000 rpm for 10 min while the supernatant was saved for the following round of virus amplification. The harvested cells underwent 4 freeze/thaw cycles and were centrifuged at 12,000 × g for 10 min to obtain cell lysates. Serial dilutions of the supernatant and cell lysates were used to transduce Ad293 cells in a 96-well plate pre-seeded with 5,000 cells overnight. The viral titers (IU/ml) were determined by counting the EGFP-positive cells under a fluorescence microscope after 30 h of culture. HUMSCs at passages 3–5 were seeded at a density of 1×10^5^ cells/well in 6-well plates. Following 24 h of culture, the medium was replaced with 1 ml of serum-free medium containing indicated adenoviruses at a multipicity of infection (MOI) of 50 for 4 h.

### Transplantation model

Ethical approval for the animal experiments was obtained from the Institutional Review Board of Shantou University Medical College. A total of 20 rats received an intraperitoneal injection of streptozotocin (STZ, Sigma-Aldrich) at 70 mg/kg in order to induce diabetes. Blood glucose levels were monitored every 3 days. Rats with blood glucose levels >16.7 mmol/l for 3 measurements were diagnosed with type 1 diabetes.

### Transplantation and physiological monitoring

The rats were divided into 3 groups, with 6–8 rats/group. After blood glucose spontaneously increased to 16.7 mmol/l, the rats were restrained and 5×10^6^ HUMSCs suspended in 0.1 ml of normal saline were injected through the tail vein. The control group underwent the same procedure, but was only injected with PBS. Body weight, blood glucose and serum insulin levels were recorded before and after cell transplantation. Blood was collected from the tail vein and blood glucose levels were measured using a blood glucose meter (Bayer, Leverkusen, Germany).

### Statistical analysis

The results are expressed as the means ± standard deviation (SD). The statistical significance of the differences was assessed by the analysis of variance. In all comparisons, a value of P<0.05 was considered to indicate a statistically significant difference.

## Results

### Characteristics of HUMSCs

**Figure 1:**
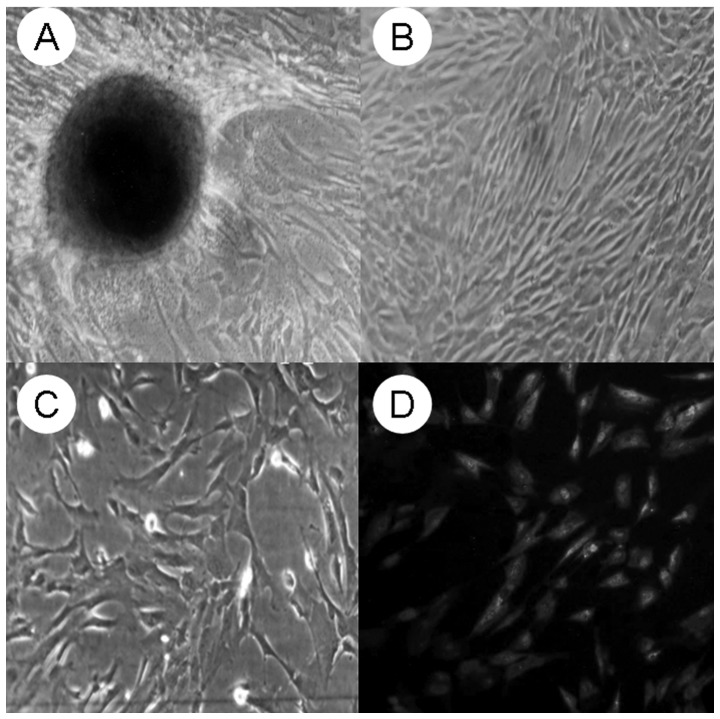
Human umbilical cord Wharton’s jelly-derived mesenchymal stem cells (HUMSCs). (A) After the Wharton’s jelly tissue sections were plated and cultured for approximately 2 weeks, some fibroblastoid cells dissociated from the tissue and adhered to the culture dish. Cells gradually multiplied and grew into a radial-like array around the adherent tissue pieces. After the HUMSCs were passaged, they showed strong proliferative ability. (B) Cells proliferated with a doubling time of approximately 24 h, but (C) this proliferation rate decreased after the 9th passage. (D) Ad293-EGFP was transfected into the HUMSCs at passages 3–5.

The HUMSCs derived from Wharton’s Jelly grew as a flat monolayer after being cultured *in vitro* for 7–10 days. After 2 weeks, some adherent cells had dissociated around the adherent tissue sections and were visible under an inverted microscope (Fig. 1A). Cells gradually multiplied and grew into a radial-like array around the adherent tissue sections. After the HUMSCs were passaged, they showed strong proliferative ability. These cells proliferated with a doubling time of approximately 24 h (Fig. 1B), but this proliferation rate decreased after the 9th passage (Fig. 1C). Ad293-EGFP was then transfected into the HUMSCs at passages 3–5 for transplantation (Fig. 1D).

### Immunological characteristics of HUMSCs

**Figure 2:**
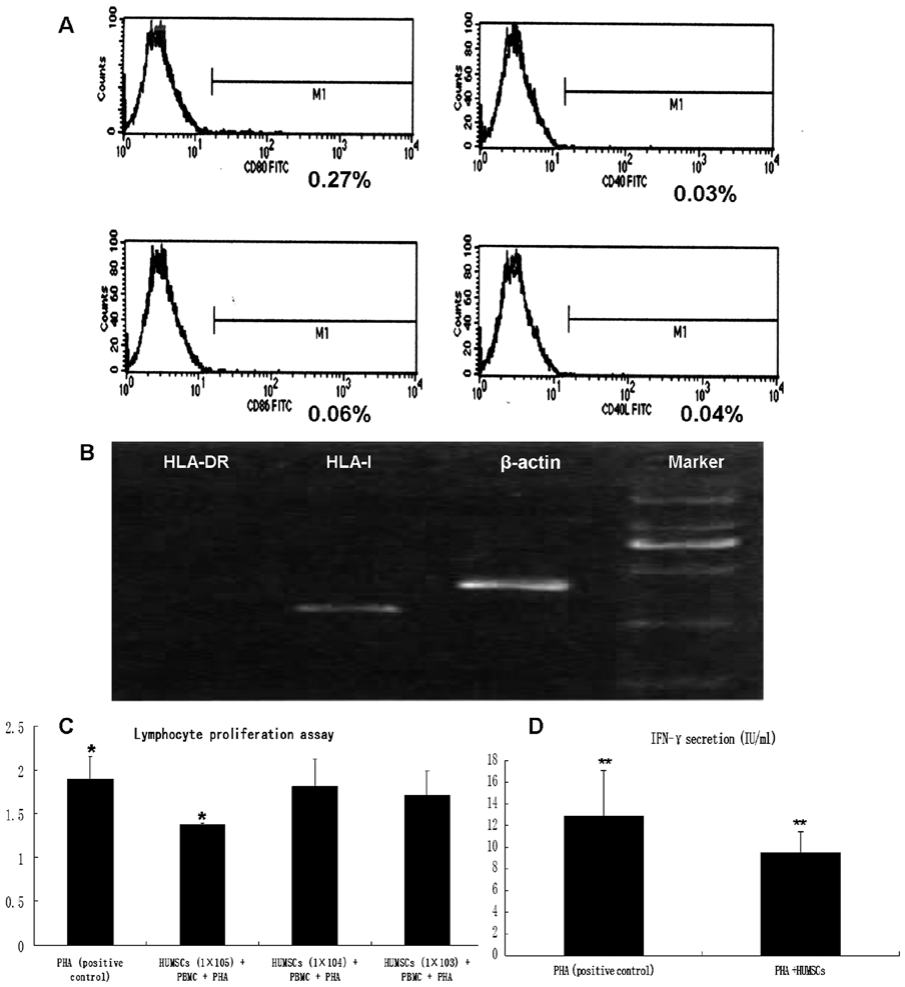
Immunological characteristics of human umbilical cord Wharton’s jelly-derived mesenchymal stem cells (HUMSCs). Cells were cultured for 3 passages, harvested and labeled with mouse anti-human monoclonal antibodies against CD40, CD40L, CD80 and CD86. (A) Flow cytometry analysis revealed low expression for all antibodies (n=3). (B) qRT-PCR analysis revealed that the cells expressed human leukocyte antigen (HLA)-I but not HLA-DR. (C) Lymphocyte proliferation assay. The CCK-8 kit was used to assess the immunomodulatory effect of HUMSCs on human peripheral blood lymphocytes (PBMCs) after phytohemagglutinin (PHA) stimulation. PBMCs + PHA (positive control), 1.90±0.25; HUMSCs (1×10^5^) + PBMCs + PHA, 1.37±0.024 (P<0.05, n=3) ^*^P<0.05. (D) ELISA of IFN-γ secretion of human PBMCs following treatment with HUMSCs. IFN-γ secretion of PBMCs was 12.88±4.22 IU/ml in the absence of HUMSCs, and decreased to 9.48±1.98 IU/ml when co-cultured with HUMSCs (P<0.05, n = 3). ^**^P<0.05.

Flow cytometry analysis revealed that the HUMSCs expressed low levels of CD80, CD86, CD40 and CD40L (Fig. 2A). qRT-PCR indicated that HUMSCs expressed the HLA-I gene (MHC-I), but not HLA-DR (MHC-II), which is closely related to graft-versus-host disease (Fig. 2B). To investigate the mechanisms responsible for the immunosuppressive effects mediated by HUMSCs, we co-cultured PHA-stimulated human PBMCs with various concentrations of HUMSC culture supernatant. The PHA-induced proliferation of human PBMCs was significantly suppressed by co-culture with different numbers of mitotically inactivated HUMSCs. The OD value of each group was as follows: PBMCs + PHA (positive control), 1.90±0.25; HUMSCs (1×10^5^) + PBMCs + PHA, 1.37±0.024 (P<0.05, n=3); HUMSCs (1×10^4^) + PBMCs + PHA, 1.81±0.31 (P>0.05, n=3); HUMSCs (1×10^3^) + PBMCs + PHA, 1.71±0.28 (P>0.05, n=3); (Fig. 2C). ELISA analysis of the PBMCs revealed that the secretion of IFN-γ was 12.88±4.22 IU/ml in the absence of HUMSCs, but decreased to 9.48±1.98 IU/ml when the cells were co-cultured with HUMSCs (P<0.05, n=3) (Fig. 2D).

### Improvement of hyperglycemia of diabetic rats and repair of damaged pancreatic cells by HUMSC transplantation

**Figure 3:**
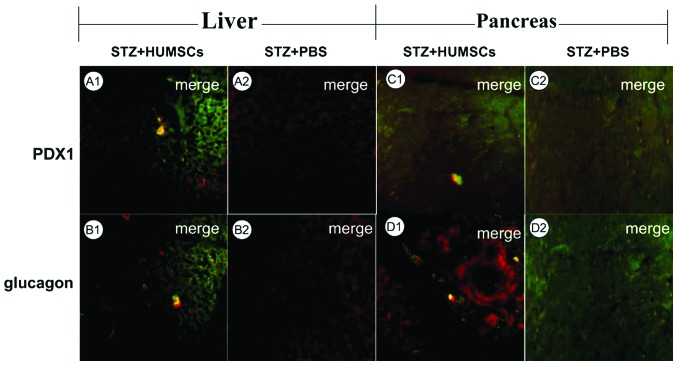
Histological analysis of the expression of human pancreatic and duodenal homeobox 1 (PDX1), glucagon and insulin in the pancreas and liver of rats with streptozotocin (STZ)-induced diabetes transplanted with human umbilical cord Wharton’s jelly-derived mesenchymal stem cells (HUMSCs) for 4 weeks. (A1, B1, C1 and D1) HUMSCs colonized in the liver and pancreas. (A1 and C1) PDX1 expression in the liver and pancreas following HUMSC transplantation. (B1 and D1) Glucagon expression in the liver and pancreas following HUMSC transplantation. (A2, B2, C2 and D2) Diabetic rats injected with phosphate-buffered saline (PBS) as the control group were negative for PDX1 and glucagon expression.

**Figure 4:**
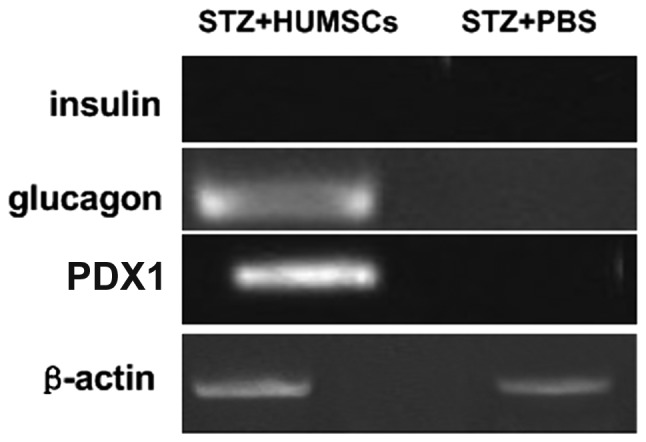
qRT-PCR analysis of human pancreatic and duodenal homeobox 1 (PDX1), glucagon and insulin mRNA expression in the pancreas of diabetic rats following human umbilical cord Wharton’s jelly-derived mesenchymal stem cell (HUMSC) transplantation for 4 weeks. The pancreas of the diabetic rats after HUMSC transplantation showed human PDX1 and glucagon expression, but no insulin expression. The control group was negative for PDX1, glucagon and insulin expression.

**Figure 5:**
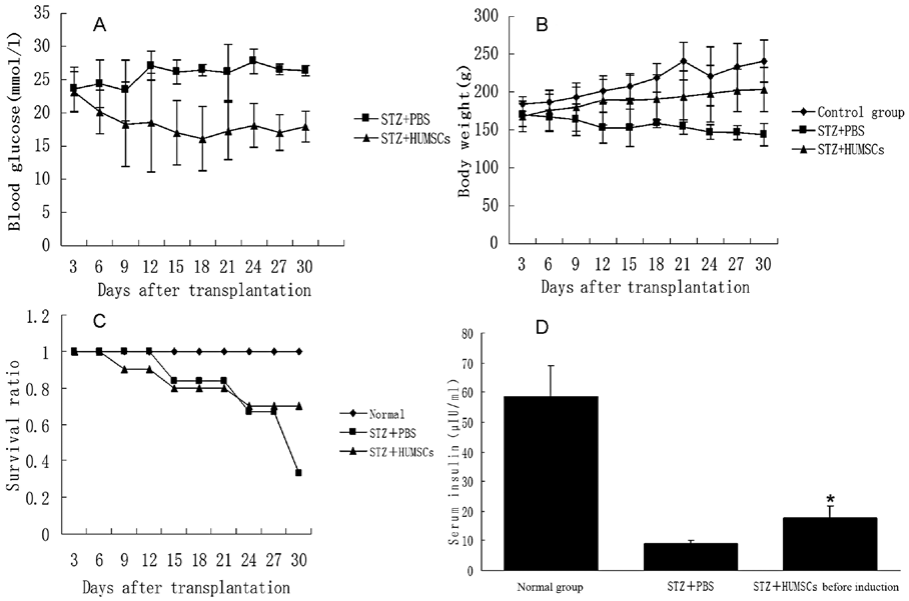
Hyperglycemia of diabetic rats following human umbilical cord Wharton’s jelly-derived mesenchymal stem cell (HUMSC) transplantation. (A) On the 9th day after transplantation, blood glucose levels decreased from 23.16±3.055 to 18.3±6.372 mmol/l compared with the control group. Afterwards, blood glucose levels were maintained between 16.1 and 18.5 mmol/l. (B) The body weight of the untreated diabetic rats decreased rapidly, whereas the body weight of the rats transplanted with HUMSCs decreased at a slower rate. (C) In addition, significant differences in survival curves and (D) serum insulin levels were observed between the control group and the group transplanted with HUMSCs. ^*^P<0.05. STZ, streptozotocin; PBS, phosphate-buffered saline.

**Figure 6:**
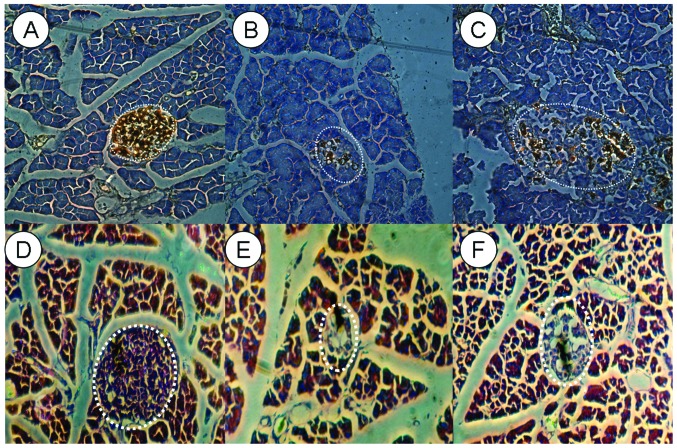
H&E and DAB staining for morphological changes of rat pancreatic islets. (A and D) The morphology of normal rat pancreatic islets showed regular arrangement, with a great quantity of cells. (B and E) Following streptozotocin (STZ) injection without any therapy, the pancreatic islets were smaller with fewer cells. (C and F) Damaged pancreatic islets of diabetic rats were partially repaired after human umbilical cord Wharton’s jelly-derived mesenchymal stem cell (HUMSC) transplantation for 4 weeks (×400).

STZ was used to induce diabetes in rats, with a single administration of 70 mg/kg. After 9 days, the blood glucose levels of the STZ-treated rats increased from the normal levels (7.14±2.08 mM) to severe hyperglycemic levels (24.04±2.84 mM). EGFP-positive HUMSCs were infused into diabetic rats on the 10th day. To avoid the aggregation of these cells and to ensure reproducible delivery, the cells were injected into the rats through the caudal vein. The rat pancreas and liver sections were analyzed by fluorescence microscopy 4 weeks after transplantation to observe whether the HUMSCs colonized *in vivo*. Green fluorescence was detected by laser scanning microscopy of frozen sections of the rat pancreas and liver. The pancreas and liver of the rats transplanted with HUMSCs stained positive for PDX1 and glucagon, but the control groups showed negative staining (Fig. 3). The PDX1 and glucagon gene were also detected in the pancreas of the group transplanted with HUMSCs, although insulin expression was not detected (Fig. 4). Blood glucose levels in the diabetic rats transplanted with HUMSCs (Fig. 5A) decreased significantly. Serum insulin levels (Fig. 5D), body weight (Fig. 5B) and the survival ratio increased (Fig. 5C), and islet repair was also improved (Fig. 6) compared with the diabetic rats not transplanted with HUMSCs.

## Discussion

MSCs can not only be derived from bone marrow, but also from blood, spleen, amniotic fluid, placenta, tendon, adipose tissue, synovial fluid, thymus cancellous bone, umbilical cord blood, skin, pulp, lungs and umbilical cord (15–18). The umbilical cord, which is considered a medical waste, can be easily obtained without adversely affecting the donor or provoking any ethical issues. Thus, it is an ideal source of cells for cell replacement therapy. MSCs derived from human umbilical cords possess self-renewal and multipotent differentiation potential. Previous studies have demonstrated that HUMSCs can be induced to differentiate into nerve cells (19), cardiomyocytes (20), pancreatic islet cells (5,21) and germ cells (22). Whether HUMSCs can colonize and differentiate into islet cells *in vivo*, and whether these are useful in the treatment of diabetes, requires further research.

Challenges with immune rejection first need to be addressed before HUMSC transplantation can be applied for the treatment of type 1 diabetes. MHC-I functions to protect MSCs from destruction by natural killer (NK) cells (23). The HLA-I antigen includes HLA-DR, HLA-DP and HLA-DQ, with HLA-DR considered the most important for allogeneic graft rejection. MHC-II can aid MSCs in escaping immune recognition by CD4^+^ T cells (13). In this study, we found that HUMSCs produce an immunosuppressive isoform of HLA-I, and do not express HLA-DR (24). This indicates that HUMSCs are a type of low immunogenic cell. Allogeneic transplant rejection is mainly mediated by recipient T cells. Recent studies have demonstrated that the excessive activation and proliferation of T lymphocytes is one of the main reasons for graft-versus-host disease (25). The full activation of naïve T cells requires the synergy of the 2 types of activation signals. When the first and second signals are bound by the corresponding ligands, T cells proliferate to form functional cell subsets. If T cells lack co-stimulatory signals, the first signal of antigen recognition is unable to effectively activate specific T cells, leading to the loss of T cell function. Thus, the synergistic activation of the stimulatory molecules is essential for normal T cell activation (26). Our results revealed that the expression of immune response-related surface antigens, such as CD40, CD40L, CD80, and CD86 is absent on HUMSCs, suggesting that HUMSCs lack the second signal system. Therefore, our results indicate that HUMSCs can escape the host immune attack *in vivo*.

In recent years, a number of studies have focused on the influence of MSC-regulated immune cells, particularly T cells. MSCs can suppress the proliferation of lymphocytes (27,28). It has been suggested that the immunomodulatory effects of MSCs are mainly exerted through the following 2 mechanisms: direct contact between MSCs and T lymphocytes, or soluble cytokines secreted by MSCs indirectly inhibiting T lymphocytes (27–29). IFN, the earliest discovered soluble cytokine, is mainly produced by activated T cells and NK cells. In a previous study, following MSC stimulation, IFN-γ secretion by Th1 cells decreased by 50%, while interleukin (IL)-4 secretion from Th2 cells increased significantly. This indicates that MSCs may induce T lymphocyte differentiation into Th2 cells. The decrease in IFN-γ secretion accompanied by the IL-4 increase can result in a decrease in the Th1/Th2 ratio (30). Our results suggested that HUMSCs (HUMSCs:PBMCs, 1:1) can inhibit the proliferation of T cells activated by PHA and that the IFN-γ secretion by T cells is decreased, indicating that HUMSCs may exert immunosuppressive effects on T cells. Taken together, our results provide important experimental evidence for the use of HUMSCs in *in vivo* transplantation.

Adult stem cells, which include HUMSCs, exhibit two important biological characteristics: first, injected adult stem cells can migrate to the target affected areas in the body after intravenous injection, and second, stem cells can be induced to differentiate into appropriate cells required for the repair of damaged tissue. This phenomenon is termed ‘site-specific differentiation’ (31). We found that HUMSCs can colonize in the liver and pancreas after transplantation via caudal veins for 4 weeks, without immune rejection. The colonized cells expressed glucagon and PDX1, which are markers of pancreatic endocrine precursor cells, but did not express insulin. These results suggest that HUMSCs can survive in different parts of the body without immune rejection, but are unable to differentiate into mature pancreatic β-cells. In addition, hyperglycemia, body weight and the survival ratio improved following the transplantation of HUMSCs into diabetic rats. On the 9th day after transplantation, blood glucose levels in the diabetic rats transplanted with HUMSCs were lower than those in diabetic rats injected with only PBS (18.3±6.372 mmol/l vs. 23.16±3.055 mmol/l, respectively). Following transplantation, blood glucose levels were maintained between 16.1 mmol/l and 18.5 mmol/l. The body weight of the diabetic rats with injected with PBS decreased rapidly, whereas the body weight of the rats transplanted with HUMSCs decreased at a slower rate. In addition, survival curves and serum insulin levels showed significant differences between the control group and the group transplanted with HUMSCs. Moreover, the pancreas of diabetic rats was repaired following HUMSC transplantation. However, the mechanisms responsble for the improvement of hyperglycemia remain unclear. We hypothesized that three possible mechanisms may be responsible for the improvement of hyperglycemia observed in this study. First, type 1 diabetes is an immune-related disease, and immune therapy can improve the symptoms of hyperglycemia. HUMSCs are a type of immunogenic cell, which also exhibit immunomodulatory functions. HUMSCs can reduce damage to islet cells by immune regulation. Second, HUMSCs are a type of support cell that may promote the repair of partially damaged islet cells to restore insulin secretion. Third, a portion of HUMSCs can be differentiated into islet cells, which secrete some insulin. However, the precise mechanism(s) involved required further investigation.

In conclusion, we found that HUMSCs did not stimulate the proliferation lymphocytes and did not induce allogeneic or xenogeneic immune cell responses. qRT-PCR revealed that the HUMSCs produced an immunosuppressive isoform of HLA-I, and did not express HLA-DR. Flow cytometry revealed that the expression of immune response-related surface antigens, such as CD40, CD40L, CD80 and CD86 was absent on the HUMSCs. These results suggest that HUMSCs may be tolerated in an allogeneic transplant. HUMSCs were transplanted into diabetic rats, and these cells survived in the liver and pancreas. The hyperglycemia of diabetic rats was improved and the damage to pancreatic cells was partly reversed by HUMSC transplantation. Hyperglycemic improvement may be related to the immunomodulatory effects of HUMSCs, although the exact mechanisms involved remain to be clarified.
